# Recognition of sepsis in primary care: a survey among GPs

**DOI:** 10.3399/bjgpopen17X100965

**Published:** 2017-05-03

**Authors:** Feike J Loots, Roeland Arpots, Rick van den Berg, Rogier M Hopstaken, Paul Giesen, Marleen Smits

**Affiliations:** 1 Research Fellow, Radboud University Medical Center, Radboud Institute for Health Sciences, Scientific Center for Quality of Healthcare (IQ Healthcare), Nijmegen, the Netherlands; 2 Researcher, Radboud University Medical Center, Radboud Institute for Health Sciences, Scientific Center for Quality of Healthcare (IQ Healthcare), Nijmegen, the Netherlands; 3 Researcher, Radboud University Medical Center, Radboud Institute for Health Sciences, Scientific Center for Quality of Healthcare (IQ healthcare), Nijmegen, The Netherlands; 4 GP, Specialist Point of Care Testing, Saltro Diagnostic Centre, Utrecht, The Netherlands; 5 GP and Senior Researcher, Radboud University Medical Center, Radboud Institute for Health Sciences, Scientific Center for Quality of Healthcare (IQ Healthcare), Nijmegen, The Netherlands; 6 Research Fellow, Radboud University Medical Center, Radboud Institute for Health Sciences, Scientific Center for Quality of Healthcare (IQ Healthcare), Nijmegen, The Netherlands

**Keywords:** sepsis, infection, general practice, primary health care, diagnosis, vital signs

## Abstract

**Background:**

Early recognition and treatment of sepsis are important to reduce morbidity and mortality. Screening tools using vital signs are effective in emergency departments. It is not known how the decision to refer a patient to the hospital with a possible serious infection is made in primary care.

**Aim:**

To gain insight into the clinical decision-making process of GPs in patients with possible sepsis infections.

**Design & setting:**

Survey among a random sample of 800 GPs in the Netherlands.

**Method:**

Quantitative questionnaire using Likert scales.

**Results:**

One hundred and sixty (20.3%) of questionnaires were eligible for analysis. Based on self-reported cases of possible serious infections, the factors most often indicated as important for the decision to refer patients to the hospital were: general appearance (94.1%), gut feeling (92.1%), history (92.0%), and physical examination (89.3%). Temperature (88.7%), heart rate (88.7%), and blood pressure (82.1%), were the most frequently measured vital signs. In general, GPs more likely referred patients in case of: altered mental status (98.7%), systolic blood pressure <100 mmHg (93.7%), unable to stand (89.3%), insufficient effect of previous antibiotic treatment (87.4%), and respiratory rate ≥22/minute (86.1%).

**Conclusion:**

The GPs' assessment of patients with possible serious infection is a complex process, in which besides checking vital signs, many other aspects of the consultation guide the decision to refer a patient to the hospital. To improve care for patients with sepsis, the diagnostic and prognostic value of assessing the vital signs and symptoms, GPs' gut feeling, and additional diagnostic tests, should be prospectively studied in the primary care setting.

## How this fits in

Sepsis is a major cause of mortality. Measurements of vital signs are successfully used in hospital settings to identify patients with possible sepsis, but it is unknown how the decision to refer a patient to hopital with a possible serious infection is made in primary care. This survey of Dutch GPs shows clinical decision making is a complex diagnostic process, in which the measurement of vital signs are not perceived as more important than various other aspects of the consultation. Although better use of the assessment of vital signs might improve the detection of sepsis, adoption of a clinical decision rule in the primary care setting only based on measuring vital signs will disregard other valuable clinical information.

## Introduction

Reducing morbidity and mortality from sepsis is a major healthcare challenge.^1^ Millions of people are suffering from sepsis each year worldwide,^2^ and one in every 20 of all deaths in England is sepsis related.^3^ Extensive efforts have been made in hospital settings, most prominently by the launching of the Surviving Sepsis Campaign (SSC) in 2004.^4,5^ Early recognition and initiation of adequate treatment are the crucial factors for successful treatment of sepsis,^6^ and screening all patients with suspected infection for 'sepsis-criteria' is important for the success of the SSC.^4,5^ In the hospital, vital signs are measured in all patients with suspected infections, and these measurements determine whether or not the patient is included in a sepsis protocol.^7^ Frequently measured vital signs in the hospital setting are the ones that are included in the systemic inflammatory response syndrome (SIRS): body temperature <36°C or >38°C, heart rate >90/minute and respiratory rate >20/minute.^7^ Recently, new consensus sepsis definitions were published, in which a new clinical decision tool was launched for bedside evaluation of patients with suspected infections: the quick Sequential Organ Failure Assessment (qSOFA).^8^ This scoring system indicates increased risk of mortality from sepsis and is positive if two out of the following three items are present: altered mental status, a respiratory rate of ≥22/minute, and a systolic blood pressure <100 mmHg.

In patients transported to an emergency department by ambulance, sepsis is frequently not detected and this is associated with increased mortality.^9–11^ More complete assessment of vital signs can improve the detection of sepsis in this setting.^9^ GPs are also encouraged to measure full sets of vital signs, to guide the decision to refer a patient to hospital with suspected sepsis.^12–14^ However, acute infections are very common in primary care, and only a small minority progress to sepsis. Screening all patients with an infection with a full set of vital signs might not be feasible, and the diagnostic and prognostic value of vital signs in this setting is unknown.

The objective of this study is to get more insight into the current clinical decision-making process of GPs in patients with acute infections, and establish how GPs use the measurement of vital signs in the decision to refer a patient to the hospital.

## Method

### Design and population

A cross-sectional questionnaire was conducted among GPs in the Netherlands. A random sample of 800 Dutch GPs was provided by the Netherlands Institute for Health Services Research (NIVEL).

### Questionnaire

The questionnaire was developed by the research team with the input from semi-structured face-to-face interviews with nine GPs, one infectiologist, one intensivist and three emergency physicians. The preparation of these interviews included a review of literature for clinical signs diagnostic for sepsis, the creation of fictitious patient cases with equivocal signs, and symptoms of sepsis. The different factors influencing the clinical decision-making process named by the interviewed professionals were categorised and included in the questionnaire. The interviews also resulted in the decision not to use the term sepsis in the questionnaire. The interpretation of the definition of sepsis was different between GPs and the interviewed specialists. GPs frequently mentioned hypotension as diagnostic for sepsis, in contrast to the other interviewed specialists who all used the SIRS criteria to diagnose sepsis. Therefore, the term 'possible serious infection' was used instead of 'suspected sepsis' to prevent misinterpretation. The questionnaire was tested in three rounds by three GPs, and feedback was obtained from the nine interviewed GPs.

In the first part of the questionnaire, the GP had to think of two adult patients from their own experience: the last patient with an acute infection that the GP had referred, and the last patient with an acute infection for whom the GP prescribed oral antibiotics. In both cases, the responders were asked whether they had measured the following vital signs: blood pressure, respiratory rate, heart rate, peripheral oxygen saturation, capillary refill time, and body temperature. Following the first case, GPs had to respond to questions concerning the importance of several aspects of the history, clinical examination and 'gut feeling' in the decision to refer the patient to the hospital. The second part of the questionnaire contained 30 general questions about the importance of history and physical examination for the decision whether or not to refer patients with a possible serious infection to the hospital. All questions were answered on a 5-point scale of importance or agreement, except the questions asking for objective information (for example, whether a certain vital sign was measured). In these cases 'yes' or 'no' options were given. Outcomes in the result section were never based on free text questions.

### Procedure

The survey was sent to 800 GPs in March 2016. GPs could return the questionnaire in print, or use a link to an electronic questionnaire in LimeSurvey (an online survey tool). A reminder was sent 2 weeks later and data collection ended after 10 weeks. Questionnaires were excluded if the background questions were not answered, or if the questionnaire was not filled in by the intended GP. Incorrect (two options ticked in the paper version) or unanswered questions were considered as missing data in the analyses. If a case did not meet the inclusion criteria (such as age <18 years), all questions concerning this case were disregarded as data. If questionnaires were undeliverable, these questionnaires were considered as not sent instead of a non-response.

### Data analysis

Data were analysed using descriptive statistics. For the clarity of the tables, the two most positive answer categories ('agree' and 'strongly agree') were combined into one category. A non-response analysis was performed based on age, sex, working area and working hours. Ninety-five per cent confidence intervals (CI) and two-sample z-tests were used to study differences between responders and the total sample. The analyses were performed using the Statistical Package for the Social Sciences version 22.0. Results were considered significant at *P*<0.05.

## Results

### Response

Of the 800 questionnaires sent, 11 were found to be incorrectly addressed. A total of 163 of the remaining 789 were completed, of which 160 (20.3%) were included and three were excluded due to incompleteness. The average age of the responders was 46.5 years and 59.4% were female. A non-response analysis showed that responders did not differ from the total sample in age, sex, and fulltime-equivalent status. Responders from strongly urban areas were, slightly underrepresented (Table 1).

**Table 1.** Characteristics of the responding GPs and total sample

**Background characteristic**	**Responders (*n* = 160),** % [95% CI]	**Total sample (*n* = 800),** % [95% CI]
**Age ≥50 years**	38.1 [31.0 to 45.8]	46.3 [42.8 to 49.7]
Female	59.4 [51.6 to 66.7]	51.8 [48.3 to 55.2]
**Working area** ^a^		
Strongly urban^b^	41.1 [33.8 to 48.9]	52.3 [48.8 to 55.7]
Moderately urban^c^	26.6 [20.3 to 34.0]	17.6 [15.1 to 20.4]
Little to non-urban	32.3 [25.5 to 39.9]	30.1 [27.0 to 33.4]
**Full-time equivalent** (FTE)^d^		
≥0.8 FTE	53.1 [45.4 to 60.7]	50.0 [46.4 to 53.6]

^a^ *n* = 158 for responders.

^b^ *P*<0.05.

^c^ *P*<0.01.

^d^ *n *= 732 for total sample.

### Cases

In the self-reported cases of patients referred to hospital, the patients had an average age of 64.3 years, and patients were mostly seen during home visits (59.2%). In the cases of patients treated with oral antibiotics, the mean age was 50.4 years and 88.3% concerned regular consultations. The respiratory tract was the most common site of infection in both the referred patients (60.3%), as well as the patients treated with antibiotics (53.9%).

Aspects of the history and physical examination that were considered most important in the cases of patients who were referred were general appearance (94.1%), gut feeling (92.1%), history (92.0%), and physical examination (89.3%) (Table 2).

**Table 2.** Importance of aspects of the history and physical examination for the clinical decision making in the self-reported cases of referred patients

**Aspect of consultation**	**Considered (very) important,** **% (*n*)**
General appearance	94.1 (143)
Gut feeling	92.1 (140)
History	92.0 (138)
Physical examination	89.3 (134)
Past illness	67.6 (102)
Age	36.2 (55)
Desire of the patient or relatives	33.8 (51)
Diagnostic tests	19.2 (29)

The vital sign measurements most frequently performed, were body temperature (88.7% in referred patients and 76.6% in patients treated with antibiotics), and heart rate (respectively 88.7% and 53.2%) (Table 3). Capillary refill time was least frequently measured (21.9% and 7.1% respectively). All vital signs were measured more frequently in the referred patients.

**Table 3.** Frequency of performed vital signs measurements in the self-reported cases of referred patients and patients treated with oral antibiotics

**Vital sign measurement**	**Referred patients, % (*n*)**	**Patients treated with antibiotics, % (*n*)**
Body temperature	88.7 (134)	76.6 (118)
Heart rate	88.7 (134)	53.2 (82)
Blood pressure	82.1 (124)	31.2 (48)
Peripheral oxygen saturation	76.8 (116)	42.2 (65)
Respiratory rate	66.2 (100)	37.0 (57)
Capillary refill time	21.9 (33)	7.1 (11)

Approximately one-quarter of the GPs expressed doubt whether or not to refer the patient in cases where they had referred, and 5.8% where unsure whether to refer patients where they had started antibiotic treatment. C-reactive protein (CRP) was available as a point-of-care (POC) test in 34.4% of the cases who were referred and in 57.1% of the cases treated with antibiotics. If POC CRP was available, it was used in 45.5% of the patients who were referred for a suspected respiratory tract infection and in 36.8% of the patients who were referred due to other infections. This corresponds to the higher prevalence of home visits in the referred group; a setting in which CRP tests are not available. In patients treated with antibiotics, the POC CRP was used in 63.3% of respiratory tract infections and 12.8% in other infections when available.

### Factors influencing referral in general

Regarding the questions whether specific premorbid conditions influenced the decision to refer a patient with a possible serious infection in general, responders agreed most on chronic use of immunosuppressive medication (96.8%) and multimorbidity (83.6%) (Table 4). The aspects of the history that were mentioned by >80% to be important were: unable to stand, insufficient effect of previous antibiotic treatment, rapid progression of illness and decreased urinary output. The three most mentioned aspects of the physical examination were altered mental status (98.7%), systolic blood pressure <100 mmHg (93.7%) and respiratory rate >22/minute (86.1%).

**Table 4.** Importance of premorbid conditions and aspects of the history and physical examination for the decision to refer a patient with a possible serious infection

**Condition**	**Considered (very) important, % (*n*)**
**Premorbid conditions**	
Chronic use of immunosuppressive medication	96.8 (154)
Multimorbidity	83.6 (133)
Diabetes	72.1 (114)
Previous hospitalisation due to infection	70.9 (112)
Congestive heart failure	68.5 (109)
Age >80 years	67.1 (106)
Lack of social support	66.7 (106)
Chronic obstructive pulmonary disease	62.2 (99)
Malignancy	55.1 (86)
Chronic use of antibiotics	52.2 (83)
Renal disease	37.1 (59)
Other heart or vascular disease	24.5 (39)
Alcohol abuse	22.6 (36)
Age >65 years	21.4 (33)
Psychiatric disorder	11.4 (18)
**History**	
Unable to stand	89.3 (142)
Insufficient effect of previous antibiotic treatment	87.4 (139)
Rapid progression of illness	83.7 (133)
Decreased urinary output	82.3 (131)
Dyspnoea	79.2 (126)
Rigors	71.1 (113)
Patient feels very ill	45.3 (71)
Decreased oral intake	28.4 (45)
**Physical examination**	
Altered mental status^a^	98.7 (157)
Systolic blood pressure <100 mmHg^a^	93.7 (148)
Respiratory rate ≥22/minute^a,b^	86.1 (136)
Sweating or clammy skin	51.3 (81)
Heart rate >90/minute^b^	47.8 (75)
Body temperature <36°C^b^	31.0 (49)
Body temperature >38°C^b^	28.3 (45)

^a^qSOFA criterium. ^b^SIRS criterium (cut-off point for respiratory rate in SIRS criteria is >20 /minute).

## Discussion

### Summary

In self-reported cases of patients referred due to a possible serious infection, body temperature, heart rate, blood pressure, and peripheral oxygen saturation were measured in the majority of the patients, but were not perceived as more important for clinical decision making than the history, general appearance, and gut feeling. In general, GPs consider the use of immunosuppressive medication, multimorbidity, and diabetes as important reasons to refer a patient to hospital with a possible serious infection. Unable to stand and insufficient effect of previous antibiotic treatment were the two most important aspects of the history for the decision to refer a patient. The individual signs of the qSOFA (altered mental status, systolic blood pressure <100 mmHg and respiratory rate ≥22/minute), were all scored (very) important for referral by the vast majority of the responders. The other signs from the SIRS criteria (body temperature <36°C or >38°, and heart rate >90/minute) were (very) important for less than half of the responders.

### Strengths and limitations

To the authors' knowledge, this is the first study on the clinical decision making of GPs in adult patients with possible serious infections, performed in a balanced, random sample of Dutch GPs across the country. The questionnaire was thoroughly designed using information from literature as well as interviews with GPs, and hospital specialists.

A limitation of the study is the rather low response rate, just above 20%. This could be due to the requested time investment of 15 minutes. Another possible explanation is that responders who started the questionnaire could not (accurately) recall the last patient they referred to hospital due to an infection, and did not complete the questionnaire. The non-response analysis, however, showed that the background characteristics of the responders did not differ from the total sample, except for the degree of urbanisation.

The frequency and relative importance of measurements of vital signs were measured, based on the last patient the responding GP had referred due to a suspected serious infection, and the last patient that the responder had treated with oral antibiotics. Thus, a sample was obtained of the two clinical scenarios and the vital signs that were measured in practice, which provides more representative results than the use of written case scenarios. However, several forms of bias could have influenced the results. Firstly, recollection of the assessment and relevance of the vital signs might not be accurate, especially when the case occurred longer than a few days before filling in the questionnaire. Secondly, recall bias of responders may have caused them not to have picked the very last case. More severely sick patients are remembered better, and more vital signs might have been assessed in these cases. Finally, GPs who perform more measurements of vital signs in general, could be more likely to complete the questionnaire. These possible forms of bias are likely to result in an overestimation of the measured vital signs. However, the relative differences between the measured vital signs will probably not be influenced by this.

### Comparison with existing literature

Although no previous research was performed on referrals by GPs of adult patients with serious infections in general, two studies were found on the clinical decision-making process in case of suspected pneumonia. Schaberg and colleagues conducted a survey among GPs, and found that in case of the clinical diagnosis of pneumonia, the presence of dyspnoea and hypotension were correlated with referral.^15^ In an observational study, Bont and colleagues investigated the prognostic accuracy of the CRB-65 score in the primary care setting.^16^ In this scoring tool, points are attributed for confusion (C), respiratory rate >30/minute (R), systolic blood pressure <90 mmHg or diastolic <60 mmHg (B), and age >65 years. In secondary care, this scoring system also includes the serum ureum (CURB-65), and is used to guide hospital admission. Bont and colleagues found that the number of points correlated with the severity of the pneumonia, but the decision to refer could not solely be based on these signs. These results are in line with the findings in the present study that hypotension, increased respiratory rate, and altered mental status are not the only important factors in the decision to refer a patient: more factors than vital signs are needed for this decision.

The results from this questionnaire containing the relative importance of comorbidity and age, can be compared to epidemiologic data from patients with sepsis reported by Henriksen and colleagues.^17^ This study showed the highest odds ratios for advanced age, immunosuppressive medication, alcohol-related conditions, and psychotic disorders. In the present study, GPs mentioned immunosuppressive medication to be most important, but they seem to underestimate advanced age, alcohol-related conditions, and psychotic disorders as risk factors for sepsis.

### Implications for further research

As timely recognition and referral of patients with sepsis is crucial, and patients are often primarily assessed in primary care, more research into the epidemiology and diagnostic strategies are needed. Identifying high risk populations is important for targeted assessment for possible sepsis. The results of this study indicate the qSOFA score is more in line with the clinical reasoning of GPs than the SIRS criteria. However, these findings cannot be used as evidence for the validity of the qSOFA for the guidance of referral. Patients who meet these criteria might not always need hospital treatment, as well as the possibility that patients with early stages of sepsis assessed by GPs might not (yet) score positive on the qSOFA.

POC testing may also provide an opportunity for improvement of the (early) detection of sepsis. CRP is a marker of infection, and is widely used as POC test by GPs to guide antibiotic prescription in patients with symptoms of respiratory tract infections.^18^ However, there is no research to support the ruling out or ruling in of sepsis with CRP, or any other biomarker in the primary care setting.

Prospective research investigating the diagnostic and prognostic value of vital signs and biomarkers should be studied in the primary care setting to guide the development of improved diagnostic algorithms. Adopting diagnostic algorithms from secondary care populations in primary care adheres to the risk of overdiagnosis and unnecessary referrals, with potential adverse effects. Algorithms may focus on patients with suspected infections or on signs of acute illness in a broader sense, and the need for hospital treatment might be more relevant than the presence of sepsis.

GPs' assessment of patients with possible serious infection is a complex process, in which in addition to the measurement of vital signs, many other aspects of the consultation guide the decision to refer a patient or not to hospital. Although better use of the assessment of vital signs might improve the detection of sepsis, adoption of a clinical decision rule in the primary care setting only based on vital signs will disregard other valuable clinical information. To improve care for patients with sepsis, the diagnostic and prognostic value of vital signs, symptoms, GPs' gut feeling, and additional diagnostic tests should be prospectively studied in the primary care setting.
